# Supplementary material for: Smooth Interpolating Curves with Local Control and Monotone Alternating Curvature
Source: Comput Graph Forum. 2022 Oct 6;41(5):25–38. doi: 10.1111/cgf.14600 (PMC9827861; doi:10.1111/cgf.14600)
Supplement: Supplementary file 1 — Supplement Material [file CGF-41-25-s001.zip › Local-Smooth-Interpolating-MonoCurvature/extern/clothoids/docs/api-matlab/program_listing_file_PolyLine.m.html]

Program Listing for File PolyLine.m — Clothoids v2.0.9

### Navigation

- index
- toc
- Clothoids »
- Program Listing for File PolyLine.m

# Program Listing for File PolyLine.m¶

↰ Return to documentation for file (`PolyLine.m`)

```
classdef PolyLine < CurveBase
  %> MATLAB class wrapper for the underlying C++ class
  methods
    %> Create a new C++ class instance for the
    %> polyline object
    %>
    %> **Usage:**
    %>
    %> \rst
    %> .. code-block:: matlab
    %>
    %>    ref = PolyLine()
    %>
    %> \endrst
    %>
    %> **On output:**
    %>
    %> - `ref`: reference handle to the object instance
    %>
    function self = PolyLine( )
      self@CurveBase( 'PolyLineMexWrapper' );
      self.objectHandle = PolyLineMexWrapper( 'new' );
    end
    % - - - - - - - - - - - - - - - - - - - - - - - - - - - - - - - - - - -
    function str = is_type( ~ )
      str = 'PolyLine';
    end
    % - - - - - - - - - - - - - - - - - - - - - - - - - - - - - - - - - - -
    %> Build a polyline object given a list of points
    %>
    %> **Usage:**
    %>
    %> \rst
    %> .. code-block:: matlab
    %>
    %>    ref.build( x, y );
    %>
    %> \endrst
    %>
    %> - `x`:  vector of x-coordinates of the points
    %> - `y`:  vector of y-coordinates of the points
    %>
    function build( self, x, y )
      PolyLineMexWrapper( 'build', self.objectHandle, x, y );
    end
    % - - - - - - - - - - - - - - - - - - - - - - - - - - - - - - - - - - -
    %> Approximate a curve of type
    %>
    %> - LineSegment
    %> - CircleArc
    %> - Biarc
    %> - ClothoidCurve
    %> - ClothoidList
    %>
    %> with a polyline
    %>
    %> **Usage:**
    %>
    %> \rst
    %> .. code-block:: matlab
    %>
    %>    ref.approx( obj, tol );
    %>
    %> \endrst
    %>
    %> - `obj`:  object storing the curve
    %> - `tol`:  tolerance admitted
    %>
    function approx( self, obj, tol )
      PolyLineMexWrapper( 'approx', ...
        self.objectHandle, obj.objectHandle, tol, obj.is_type() ...
      );
    end
    % - - - - - - - - - - - - - - - - - - - - - - - - - - - - - - - - - - -
    %>
    %> Return the points of the polyline (the polygon)
    %>
    function [ x, y ] = polygon( self )
      [ x, y ] = PolyLineMexWrapper( 'polygon', self.objectHandle );
    end
    % - - - - - - - - - - - - - - - - - - - - - - - - - - - - - - - - - - -
    %>
    %> Return the index of the segment containing `s` curvilinear abscissa
    %>
    function idx = s_to_index( self, s )
      idx = PolyLineMexWrapper( 's_to_index', self.objectHandle, s );
    end
    % - - - - - - - - - - - - - - - - - - - - - - - - - - - - - - - - - - -
    %> Plot the polyline
    %>
    %> **Usage:**
    %>
    %> \rst
    %> .. code-block:: matlab
    %>
    %>   ref.plot();
    %>
    %>   fmt1 = {'Color','blue','Linewidth',2}; % first arc of the biarc
    %>   fmt2 = {'Color','red','Linewidth',2};  % second arc of the biarc
    %>   ref.plot( fmt1, fmt2 );
    %>
    %> \endrst
    %>
    %> - `fmt1`: format of the odd segment
    %> - `fmt2`: format of the even segment
    %>
    function plot( self, varargin )
      if nargin > 1
        fmt1 = varargin{1};
      else
        fmt1 = {'Color','red','LineWidth',3};
      end
      if nargin > 2
        fmt2 = varargin{2};
      else
        fmt2 = {'Color','blue','LineWidth',3};
      end
      [ x, y ] = self.polygon();
      for k=2:length(x)
        if mod(k,2) == 0
          plot( x(k-1:k), y(k-1:k), fmt1{:} );
        else
          plot( x(k-1:k), y(k-1:k), fmt2{:} );
        end
        hold on;
      end
    end
  end
end
```

### Quick search

### Table of Contents

- Matlab Interface Manual
- C++ API
- MATLAB API

«
hide menu

menu
sidebar
»

### Navigation

- index
- toc
- Clothoids »
- Program Listing for File PolyLine.m

© Copyright 2021, Enrico Bertolazzi and Marco Frego.
Created using Sphinx 4.2.0.
